# Error awareness and the insula: links to neurological and psychiatric diseases

**DOI:** 10.3389/fnhum.2013.00014

**Published:** 2013-02-04

**Authors:** Tilmann A. Klein, Markus Ullsperger, Claudia Danielmeier

**Affiliations:** ^1^Department of Neurology, Max Planck Institute for Human Cognitive and Brain SciencesLeipzig, Germany; ^2^Day Care Clinic for Cognitive Neurology, University Clinic LeipzigLeipzig, Germany; ^3^Donders Institute for Brain, Cognition, and Behaviour, Radboud University NijmegenNijmegen, Netherlands; ^4^Institute of Psychology, Otto-von-Guericke University MagdeburgMagdeburg, Germany; ^5^Center for Behavioral Brain SciencesMagdeburg, Germany

**Keywords:** insula, error awareness, anosognosia, lack of insight, conscious error perception, error-related negativity (ERN), error positivity (Pe)

## Abstract

Becoming aware of errors that one has committed might be crucial for strategic behavioral and neuronal adjustments to avoid similar errors in the future. This review addresses conscious error perception (“error awareness”) in healthy subjects as well as the relationship between error awareness and neurological and psychiatric diseases. We first discuss the main findings on error awareness in healthy subjects. A brain region, that appears consistently involved in error awareness processes, is the insula, which also provides a link to the clinical conditions reviewed here. Then we focus on a neurological condition whose core element is an impaired awareness for neurological consequences of a disease: anosognosia for hemiplegia (AHP). The insular cortex has been implicated in both error awareness and AHP, with anterior insular regions being involved in conscious error processing and more posterior areas being related to AHP. In addition to cytoarchitectonic and connectivity data, this reflects a functional and structural gradient within the insula from anterior to posterior. Furthermore, studies dealing with error awareness and lack of insight in a number of psychiatric diseases are reported. Especially in schizophrenia, attention-deficit hyperactivity disorder, (ADHD) and autism spectrum disorders (ASD) the performance monitoring system seems impaired, thus conscious error perception might be altered.

## Introduction

For daily life it is important that we become aware of the consequences of our actions, of failures and limitations that force us to change our behavior and strategies. In clinical settings, reduced conscious perception of errors has been associated with poor insight in consequences of neurological conditions (O'Keeffe et al., 2004). Whereas it is still unclear whether conscious perception of errors is a necessary prerequisite for all kinds of post-error adjustment (cf. Danielmeier and Ullsperger, 2011), in situations when several people work together it certainly is, because only after conscious detection and appreciation of an error it can be communicated to others and appropriate measures can be taken. This review deals with brain areas that have been shown to play a role in conscious error detection (or “error awareness”) in functional magnetic resonance imaging (fMRI) or patient studies. Additionally, electrophysiological studies addressing error awareness and their functional and clinical relevance will be discussed.

Relevant brain areas in the context of error awareness are the posterior medial frontal cortex (pMFC), the thalamus and, as we want to argue in the course of this review, most important, the anterior insula. The insula seems to be crucial for error awareness, because fMRI studies revealed that the insula is consistently activated for consciously perceived errors compared to unperceived errors (Klein et al., 2007a; Hester et al., 2009). Recently, the insula has been suggested to be of relevance for interoception (Craig, 2009, 2011). On the one hand, interoception might contribute to conscious error detection processes, because errors elicit a number of autonomic responses, e.g., changes in heart rate (Wessel et al., 2011) and skin conductance responses (O'Connell et al., 2007), that could potentially be detected by the (anterior) insula. On the other hand, lesions in more posterior regions of the insula have been associated with anosognosia for hemiplegia (AHP, Karnath et al., 2005). AHP describes the unawareness of motor deficits that are related to hemiplegia. Vocat and Vuilleumier (2010) proposed that anosognosia is a multi-componential disorder affecting bodily awareness (amongst other things), or in other words, affecting interoception. Thus, both error awareness and AHP might be linked through interoception or the proper integration of interoception and exteroception. The potential relationship between error awareness and AHP has already been discussed by Vocat and Vuilleumier (2010). Since error awareness processes have been located in the inferior anterior part of the insula, and AHP can be observed after lesions in more posterior parts of the insula, we propose that there is a functional gradient in the insula from anterior to posterior that reflects different aspects of interoception. A similar gradient has also been observed in cytoarchitectonics and structural as well as functional connectivity analyses of the insular cortex. In this review we want to argue that the insular cortex, due to its cytoarchitectonic layout and its functional as well as structural connectivity, is perfectly suited to play a key role in error awareness. The processing of interoceptive information might deliver information that supports error awareness. The recently proposed role of the insula as a relay station regulating interactions between brain networks involved in external attention and interoceptive cognition (Menon and Uddin, 2010) fits well with the proposed role of the insula in error awareness. Interoceptive information supports error awareness, which in turn might lead to an orienting reaction to the now salient external event.

In the following, we will start with a brief overview over the research on error awareness and its electrophysiological correlates. Then, we will report the neuroanatomical and neurochemical basis of error awareness, with a special focus on the insular cortex. The insular focus and the concept of interoception will lead to a brief discussion on AHP. To complete the picture on error awareness, we selectively report findings on those psychiatric disorders where (a) structural or functional changes in the insula have been reported (among other changes in various brain areas), and (b) electrophysiological studies on error processing exist that suggest an impairment in error awareness.

Error awareness describes the ability to consciously perceive one's own mistakes. A mistake is the failure to achieve the intended goal of an action. Current views suggest that error awareness can be explained by an accumulating evidence account (Ullsperger et al., 2010; Vocat and Vuilleumier, 2010; Wessel et al., 2011; Wessel, 2012). This account describes the accumulation of evidence for an error from very different sources, e.g., pMFC activity, proprioceptive and other sensory input that deviates from expectation, and/or changes in the autonomic nervous system. Thus, each event is evaluated as to whether it indicates or predicts an action outcome that is different (worse) than intended. For example, a deviation of the motor efference copy and/or the proprioceptive and sensory feedback from predictions made in forward models of the action (Desmurget and Grafton, 2000) can indicate that the entire action is going to fail. Later, the observation of the outcome itself deviating from the goal provides additional evidence for the mistake. Moreover, when two alternative response tendencies compete, the resulting response conflict has been suggested to provide evidence for the error (Yeung et al., 2004). These pieces of evidence, which by themselves can be expressed as deviations from predictions (prediction errors), accumulate during and after the action. Evidence accumulation can start as early as the action is initiated, but the point of awareness can be temporally detached from the actual error (e.g., in underdetermined responding, error awareness can only occur after external feedback). Vocat and Vuilleumier (2010) suggest a comparable mechanism, for explicit awareness of motor impairments, i.e., the integration of information from different channels.

It should be noted that the evidence accumulation account outlined above is compatible with predictive coding accounts of awareness and motor action control (e.g., Friston et al., 2010; Seth et al., 2011). Whether error awareness itself is a product of another higher-level predictive-coding mechanism that, for example, compares the predicted task performance with the accumulating prediction error evidence remains to be investigated.

Reduced error awareness can occur under normal as well as pathological conditions. One major determinant may be the type of error that is committed. Depending on the complexity of the task, the level of processing and the information available, different error types can be detected with different reliability (Reason, 1990). During action slips and lapses that occur during skill-based, routine behavior usually all information to detect the error is available such that almost all errors are consciously perceived. For example, in speeded choice reaction time tasks, such as the Eriksen Flanker task, where subjects have to respond to a centrally presented target stimulus and ignore (conflicting) stimuli next to the target, usually 90% or more errors are detected by healthy participants (Ullsperger and Von Cramon, 2006; Seifert et al., 2011). In contrast, mistakes of planning or judgment during rule-based or knowledge-based behavior are less easy to detect (Reason, 1990). Particularly, if errors result from failures of interpretation and comprehension of the current task situation, they are often performed with high confidence and are therefore often missed. In underdetermined, overwhelmingly complex situations, participants have a low confidence in their responses, but without feedback they are unable to determine whether their response was correct or erroneous. Errors can also result from insufficient perceptual information, for instance, when stimuli are degraded or masked. In this case, the necessary sensory information for performance monitoring processes is missing, so that errors cannot be noticed. If errors result from general decreases of arousal and a disengagement from the task (Eichele et al., 2008), their likelihood to be consciously perceived can be expected to decrease. This may be particularly true for errors that occur after sleep deprivation (Scheffers et al., 1999; Chee et al., 2008), but this hypothesis still needs to be tested. Indeed Shalgi et al. (2007) were able to show that greater task monotony (presumably via reduced arousal) reduces the number of errors that are consciously perceived. Finally errors can result from failures in the processing of the perceptual properties of the stimulus (see also section “Experimental Paradigms to Investigate Error Awareness”).

Usually, error awareness has been studied by asking participants whether they noticed having made a mistake, since it has been unclear whether error awareness can be quantified reliably in a more direct and objective way, i.e., without asking participants after every trial. However, recent studies suggest that the amplitude of the error positivity (Pe) might be a good quantitative correlate of error awareness (Murphy et al., 2012; see below), particularly when quantified in single trials and/or time-locked to the error-signaling response (see below), since the Pe seems to be linked to the time when the subject presses the error-signaling button.

Often, participants are asked to signal any encountered error by pressing an “error signaling button” (Rabbitt, 1968). This procedure may, however, induce some response bias, because for responses considered correct no motor response is needed. Furthermore, short inter-trial intervals may prevent participants from signaling errors despite being aware of them. A number of studies therefore explicitly asked participants after each trial, whether they deemed the preceding behavior correct or incorrect (Endrass et al., 2007; Klein et al., 2007a; Logan and Crump, 2010; Wessel et al., 2011).

## Experimental paradigms to investigate error awareness

Three kinds of tasks have been used to study error awareness. As discussed in Ullsperger et al. (2010), they appear to interfere with the accumulation of error evidence at different stages, thereby resulting in a sufficient number of errors that remain unconscious. (1) When the detection of stimuli is rendered increasingly difficult, for example by degrading visibility (Scheffers and Coles, 2000) or metacontrast masking (Maier et al., 2008; Cohen et al., 2009; Steinhauser and Yeung, 2010), participants not only make more errors, they are also less certain about their performance and miss a number of mistakes. (2) Oculomotor tasks, such as the antisaccade task, have been very successful in inducing unperceived errors (Nieuwenhuis et al., 2001; Endrass et al., 2007; Klein et al., 2007a; Wessel et al., 2011). It appears that error evidence from proprioception and sensory (visual) input is rather weak for short and immediately corrected prosaccades, such that they are easily overlooked (Ullsperger et al., 2010). (3) In complex task sets consisting of a number of competing and constantly to-be-monitored rules, some rule representation may be dominant and others only weakly represented. Errors related to one rule may then remain undetected more frequently. This principle has been successfully applied in a number of studies using a Go/NoGo task with two different NoGo conditions (Hester et al., 2005; O'Connell et al., 2007). The typical error awareness task in these studies consisted of color words printed in congruent or incongruent ink (as in a Stroop task). The majority of stimuli were congruent words, serving as signal for a Go response. In contrast, when incongruent stimuli appeared (rule 1) or a color word was repeated in two successive trials (rule 2), subjects had to withhold their response (NoGo). Continuously monitoring both congruency and repetitions appears to be difficult and leads to many NoGo errors that subjects are not aware of.

## Electrophysiological correlates of error awareness

Performance monitoring is associated with a number of neural correlates that appear to be differentially modulated by conscious error perception. Based on early findings in antisaccade tasks (Nieuwenhuis et al., 2001; Klein et al., 2007a) and the Go/NoGo “error awareness task” (O'Connell et al., 2007) it was assumed for a long time that the error-related negativity (ERN) (Falkenstein et al., 1990; Gehring et al., 1993), a frontocentral event-related potential occurring shortly after erroneous button presses in speeded choice reaction time tasks, was present on all error trials and unaffected by conscious error perception. In contrast, the later and more posterior Pe (Falkenstein et al., 1990) was present only when errors were perceived consciously (Nieuwenhuis et al., 2001; Endrass et al., 2007). Similarly, neuroimaging studies seemed to suggest that the pMFC, the putative generator of the ERN, was active on both reported and unreported errors, whereas the anterior insula was specifically modulated by error awareness (Ullsperger et al., 2010).

However, a recent study using an antisaccade task (Wessel et al., 2011) as well as studies using degraded or masked stimuli (Scheffers and Coles, 2000; Steinhauser and Yeung, 2010) showed that the ERN may co-vary with error awareness as well. Smaller ERN amplitudes are associated with a lower likelihood to consciously perceive the error. Shalgi and Deouell (2012) were able to show that the amplitude of the ERN is related to error awareness and that it co-varies with the individual confidence with which an answer was made (higher amplitude in aware errors for confident subjects). In line with this, more recent fMRI studies reported greater pMFC activity in aware compared to unaware errors (Hester et al., 2009, 2012; Orr and Hester, 2012; see also “Posterior Medial Frontal Cortex”). Current views suggest that the ERN (and feedback-related negativity, FRN) reflects the processing of single pieces of objective evidence for an error (or other events requiring adaptation). For example, when stimulus-induced evidence is low, the ERN amplitude is low (Scheffers and Coles, 2000). In a flanker task study with response feedback, in most trials feedback is redundant and not associated with an additional negativity (De Bruijn et al., 2004; Gentsch et al., 2009). When, for any reason on some trials efference copy or perceptual information available at the time of the response was reduced (behaviorally reflected in prolonged remedial action times), not only the ERN was reduced in amplitude but also an FRN appeared in the same trial (Gentsch et al., 2009). Thus, the additional feedback information was used to disambiguate the situation. In such trials, two small pieces of evidence for an error occurred in short succession and were both reflected in medial frontal negativities, namely the (reduced) ERN and (increased) FRN. This is compatible with the view that error evidence accumulates with new incoming information related to action outcome. When sufficient evidence has accumulated, this may be the basis of error awareness. In contrast to the ERN, the Pe reflects the subjective (accumulated) evidence associated with conscious awareness (cf. Wessel, 2012). A recent study suggests that the Pe amplitude and latency correlates with the subject's indication of error awareness and predicts reliably whether an error would be consciously perceived or not (Murphy et al., 2012). Thus, the Pe appears to be a good measure of error awareness. Murphy et al. (2012), however, suggest investigating the Pe locked to the error-signaling response and not time-locked to the response. This should make clear that a reduced amplitude is really due to diminished awareness and not to for example a higher variability in the latency of error awareness.

## Functions of the insular cortex

**Figure 1:**
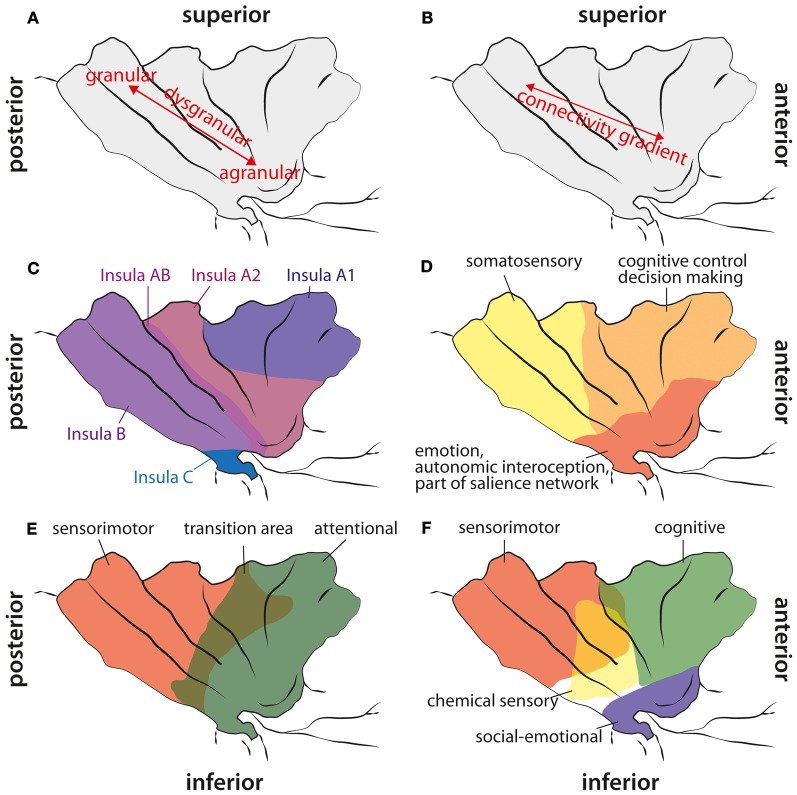
**Cytoarchitectonic, structural connectivity, and functional maps of the human insula. (A)** Cytoarchitectonic gradient from agranular cortex in the anterior inferior insula via dysgranular cortex to granular cortex in the posterior part of the insula. **(B)** Structural connectivity gradient in the insula according to Cerliani et al. (2012). Unlike in other brain areas (e.g., premotor cortex), they did not find any clear border between insula regions based on the structural connectivity profile; instead, they reported a gradual change in connectivity patterns from anterior to posterior insular areas. **(C)** Cytoarchitectonic map adapted from Von Economo and Koskinas (1925). They did not find any agranular areas within the insula (except from a fronto-insular region anterior to what is shown here), but a less granulated area “Insula A1” and stronger granulated areas more posterior. Note, that they explicitly report a transition area “Insula AB” between anterior and posterior insular regions. **(D)** Functional areas of the insula according to Deen et al. (2011). **(E)** Functional differentiation of the insula according to Cauda et al. (2011). Note, that they report a transition area between the anterior and the posterior part. **(F)** Functional areas in the insula according to Kurth et al. (2010). They reported four different areas, also with a clear overlap in the middle aspects of the insula.

Several reviews about the functional neuroanatomy of the insula have been published recently (Kurth et al., 2010; Menon and Uddin, 2010; Cauda et al., 2011; Kelly et al., 2012). Therefore, we only want to give a brief overview over functions that have been associated with this brain area (see Figure 1). In line with the cytoarchitechtonic gradient in the insula (Mesulam and Mufson, 1982a; see below)—from agranular cortex in the (inferior) anterior part to dysgranular cortex in the middle part to granular cortex in the posterior part—Cauda et al. (2011) reported two overlapping functional networks, an attention-related network anterior, and a sensorimotor network posterior, with a large overlap of both networks in mid-insula areas. By means of a meta-analysis of functional neuroimaging data, Kurth et al. (2010) found four distinct functional regions within the insula. They described the inferior anterior part of the insula in terms of social-emotional processes, the superior anterior part in relation to cognitive processes, a chemical sensory area in the middle part and a sensorimotor area in the posterior part, with considerable overlap between functional areas especially in the middle part of the insula. Based on resting state data, Kelly et al. (2012) reported up to nine different functional clusters within the insula, also with considerable overlap between these clusters. In agreement with other studies, they found cognitive and attentional processes to be located in more anterior parts, emotional aspects in inferior parts, and sensorimotor and interoceptive processes in posterior parts. Additionally, Mutschler et al. (2009) reported consistent activation of the inferior anterior insula in relation to peripheral physiological changes. As reviewed already by Augustine (1996), the insula is engaged in a wide variety of functions, such as visceral sensory and motor processes, vestibular processes, limbic integration, motor association, and language-related auditory processing. In the last decade, the role of the insula in interoception has been emphasized, as well as its role in emotional and interoceptive awareness or awareness in general (Critchley et al., 2004; Craig, 2011; Simmons et al., 2012). Recently, it has been suggested that the right fronto-insular cortex plays a crucial role in switching activity between different functional networks, especially the default mode and an executive network (Sridharan et al., 2008), or that the anterior insula is involved in detecting novel salient stimuli in different modalities (Menon and Uddin, 2010). This last hypothesis is in agreement with the suggestion that the anterior insular cortex (AIC) is part of a salience network, consisting of the AIC, the anterior cingulate cortex, the amygdala, and the inferior frontal gyrus (IFG) (Seth et al., 2011). The notion that the AIC belongs to a salience network fits well with observations that the AIC plays a crucial role in error awareness (e.g., Klein et al., 2007a; see below), because consciously perceived errors are obviously salient events, whereas unnoticed errors are not. Furthermore, there are strong intra-insular connections (Augustine, 1996; Kurth et al., 2010), suggesting that posterior parts might feed information into the salience network located in AIC. An interruption of this process due to lesions within the insula might result in a mismatch in bodily or sensorimotor perceptions. Especially the awareness for limb functioning and the sense for limb ownership seem to require intact insular functions. As pointed out by Karnath et al. (Karnath et al., 2005; Baier and Karnath, 2008; Karnath and Baier, 2010), especially the right posterior insular was repeatedly found in lesion analysis studies with stroke patients to be a central element in the process of interoceptive awareness necessary for intact sense of limb functioning and limb ownership. Berti et al. (2005) also report that, besides lesions in motor and premotor areas, lesions to prefrontal areas like BA 46 and the insula are differentially involved in AHP as well (but less frequent). More recently, however, Vocat et al. (2010) reported lesions to the anterior insula as being crucial for AHP especially during the hyperacute (three days post insult) phase.

## Neuroanatomical basis of error awareness

A few brain areas have been associated with conscious error perception. Most studies suggest that the anterior insula is crucial for error awareness. Besides the insula, the pMFC (comprising the pre-supplementary motor area and an area that is equivalent to the ACC in monkeys, i.e., the anterior mid-cingulate cortex, aMCC; cf. Vogt, 2005), and the thalamus might be important for error awareness. In the majority of studies reporting insula activations, the aMCC is co-activated with the insula (cf. Craig, 2009). In the following, insula anatomy and connectivity will be described briefly. Then, we will report studies providing evidence that the anterior insula, the thalamus, and the pMFC are crucial for conscious error perception.

### Insula: structure and connectivity

The anterior inferior part of the human insula consists of agranular cortex. Specific cytoarchitectonic areas of the insula are preferentially connected to cytoarchitectonically similar areas in other parts of the brain (Mesulam and Mufson, 1982b; Mufson and Mesulam, 1982), that is, agranular areas are predominantly connected to other agranular cortical areas, e.g., the anterior cingulate cortex. Cytoarchitectonically, there is a gradient from agranular cortex, located in the anterior insula, to dysgranular cortex located in the middle part of the insula and to granular cortex in the posterior insula (see Figure 1). However, Von Economo and Koskinas (1925) doubted that there are agranular areas in the insula (except of a fronto-insular transition area at the anterior border of the insula), but their data also suggest the presence of a cytoarchitectonic gradient from dysgranular cortex in the superior anterior insula to granular cortex in posterior insula regions.

The von Economo neurons (VENs) have been found in both the anterior cingulate cortex and the frontal insular cortex in humans and great apes (Von Economo, 1926; Allman et al., 2010; Seeley et al., 2012), and recently also in macaque monkeys (Evrard et al., 2012). They appear to be projection neurons and most likely project to the frontal pole, other frontal, and insular areas, the septum, and the amygdala (Allman et al., 2010). Allman et al. (2010) found that the protein, which is encoded by the DISC1 gene (disrupted in schizophrenia), is preferentially expressed in VENs, thereby relating these neurons to a genetic basis of schizophrenia.

In macaque monkeys the insular cortex is characterized by widespread anatomical connections (for an overview see Cerliani et al., 2012), among them projections to autonomic nuclei in the brainstem and several thalamic nuclei. Cerliani et al. (2012) investigated white matter connections of the insula cortex in humans by using diffusion-weighted imaging (DWI). This type of connectivity data can be used to parcellate brain regions according to their connectivity profile. Usually, at the border between two functionally different regions, a clear change in the connectivity profile can be observed. However, Cerliani et al. (2012) reported that this connectivity-based parcellation did not yield reliable, clearly distinguishable subregions for the insula, since they did not find any of these abrupt changes in the connectivity profile of the insula. The authors instead argue that their connectivity data suggest a gradient in connectivity profiles from the anterior to posterior insula, which show a large overlap in their connectivity profile without any distinct borders. According to their results, the anterior (agranular) part of the insula is mainly connected to the orbitofrontal cortex, the amygdala, and the rostral part of the IFG, whereas the posterior (granular) part of the insula is connected to parietal and posterior temporal areas, caudal parts of the IFG, and the lateral premotor cortex. The dysgranular insular cortex in between shows some overlap in the connectivity pattern with both the anterior and posterior insula. While the anterior dysgranular areas are more similar to the connectivity pattern of the agranular insula, the posterior dysgranular areas are more similar to the connectivity pattern of the granular insula. Thus, in line with the cytoarchitectonic gradient from agranular to granular cortex, there also is a connectivity gradient from anterior to posterior in the insula without any distinct borders that could potentially have been defined based on abrupt changes in connectivity (Cerliani et al., 2012). However, one limitation of this study is that only brain areas, that are part of the probabilistic cytoarchitectonic map from Juelich (cf. Cerliani et al., 2012 for a complete overview of used maps), have been included as potential target areas. Therefore, some brain areas, such as the aMCC, that are connected to the insula in rhesus monkeys (Morecraft et al., 2012), but which are not yet included in these maps, could not be found as projection targets of the insula. The large diversity of insula connections makes this brain area well suited for the integration of external signals with interoception (Seth et al., 2011).

Cauda et al. (2011) used resting state fMRI measures to identify functional networks of the insula. They found an anterior and a posterior network in the insula and a transition area in between, thus, corroborating the insular gradient in the cytoarchitectonic data and the DWI study by Cerliani et al. (2012). The anterior insula was functionally connected to the rostral ACC, middle and inferior lateral frontal cortex and temporoparietal areas. The posterior insula was part of a network consisting of the superior posterior cingulate cortex, motor areas (including pre-SMA and premotor cortex), somatosensory areas, temporal cortex, and parts of the occipital lobe. The authors described the anterior insular network as “attention network” and the posterior insular network as “sensorimotor network.”

Deen et al. (2011) divided the insula in 3 subregions based on functional connectivity measures: an anterior inferior part, an anterior superior part, and a posterior part. The inferior part of the anterior insula was most strongly connected to the pregenual ACC, while the superior part of the anterior insula was mainly connected to the aMCC. The posterior insula was functionally connected to posterior MCC. The results by Deen et al. (2011) suggest that areas of the insula are systematically connected to the medial frontal cortex (MFC) with more anterior insular regions being connected to more anterior MFC regions and more posterior insular regions to more posterior MFC areas. Besides the functional connections between insula and MFC, extensive connectivity with other brain areas were reported (Deen et al., 2011): the inferior anterior insula was connected to opercular cortex, the posterior IFG, and the superior temporal sulcus. The superior anterior insula showed functional connections with visual areas, the medial thalamus, opercular and posterior orbitofrontal cortex, pre-supplementary motor cortex, precentral sulcus, supramarginal gyrus, and the anterior IFG. The posterior insula was connected with motor (including SMA) and somatosensory areas, opercular cortex, pre-SMA, and the medial thalamus. All insular subregions were interconnected, providing a structural basis for the communication between different parts of the insula, i.e., between somatosensory and attention-related areas.

Co-activations of brain regions, and thus potential functional networks, can also be demonstrated in fMRI meta-analysis as well as in spatial independent component analysis (ICA) of fMRI data. A meta-analysis of performance monitoring showed co-activation of anterior insula, aMCC, and thalamic regions (Klein et al., 2007a; Ullsperger et al., 2010). Similarly, the posterior MFC, thalamus, and anterior insula were repeatedly covered by the same independent components, suggesting a highly similar signal time course in these regions (Dosenbach et al., 2007; Eichele et al., 2008; Danielmeier et al., 2011).

In conclusion, the insular cortex is involved in at least 2–3 functional networks. Both macaque cytoarchitectonics and human connectivity studies (Cauda et al., 2011; Deen et al., 2011) suggest that there might be no distinct subdivisions in the insula, but instead, that there is an anterior-to-posterior gradient in both cytoarchitectonics and connectivity. Craig (2011) suggested a functional gradient within the insula, with posterior insula regions reflecting the objective stimulation strength (e.g., of painful stimuli), and anterior regions reflecting subjective feelings related to this stimulus. This suggestion is in line with interoceptive processes that have been associated with the insula.

### The insula and awareness deficits

Especially the anterior inferior insula seems to be involved in error awareness. In an antisaccade task, the anterior insula was the only brain area distinguishing between consciously perceived and unperceived errors (Klein et al., 2007a). In an fMRI study employing the error awareness Go/NoGo task described above, it has been shown that consciously perceived errors go along with larger BOLD responses in the right insula and in left inferior parietal regions (Hester et al., 2009).

According to Kurth et al. (2010), the anterior insula is functionally related to attentional and cognitive processes and autonomic responses. A meta-analysis (Mutschler et al., 2009) has associated the anterior inferior insula with autonomic responses, such as changes in heart rate or skin conductance rate. Furthermore, this part of the insula is often co-activated with the amygdala. Intraoperative stimulations of the insula in epileptic patients led to changes in cardiac responses (Oppenheimer et al., 1992). Wessel et al. (2011) have recently described a link between error awareness and cardiac responses. Following perceived errors, a stronger heart beat deceleration was observed compared to unperceived errors. Craig (2009) suggested that the right AIC activity correlates with subjective feelings of body states, e.g., pain or awareness of heartbeats. Similarly, Paulus et al. (2009) suggested that the functional role of the insula is to detect discrepancies between the predicted body state and the actual body state. Recently, Seth et al. (2011) proposed a model of awareness (or “presence”) in general, i.e., not restricted to error awareness. They suggested that the insula is crucial for the integration of interoceptive and exteroceptive signals, and the anterior insula is assumed to be a “comparator or error module” (Seth et al., 2011). This is likely to also apply to error awareness, as already discussed in the Introduction. However, at this point it is unclear whether the autonomic response is cause, result or correlate of error awareness.

As mentioned above, the posterior region of the insula is connected to the SMA and premotor areas (Cerliani et al., 2012). Lesions in the right posterior insula can lead to AHP (Karnath et al., 2005), which can be defined as selective disorder of awareness for motor deficits (Spinazzola et al., 2008). This supports the notion by Craig (2009) that the insular cortex in general is related to awareness. While the anterior insula has been associated with error awareness, the middle and posterior insular cortex seem to be associated with the awareness of motor and somatosensory processes (Karnath et al., 2005; Spinazzola et al., 2008). Thus, depending on the exact lesion location within the insula, one might observe different, domain-specific awareness deficits. This gradient in awareness deficits from anterior to posterior insular areas might reflect the underlying connectivity and cytoarchitectonics gradient within the insula.

### Thalamus and awareness deficits

Some studies suggest that thalamic lesions can also impair error awareness and lead to anosognosia (De Witte et al., 2011; Peterburs et al., 2011; Seifert et al., 2011). In a review by De Witte et al. (2011), two patients were described who suffered from anosognosia after bilateral thalamic lesions. In a study by Seifert et al. (2011), patients with chronic thalamic lesions were asked to participate in a flanker task and signal their errors with a button press. While the age matched control group signaled 85% of their errors on average, the patient group indicated only 39% of their erroneous responses. This suggests that in patients, suffering from thalamic lesions, a majority of errors is not perceived consciously. This result has been replicated in an antisaccade task (Peterburs et al., 2011). Patients with thalamic lesions signaled their errors significantly less often than the healthy control group. Thus, there is preliminary evidence that error awareness is reduced following thalamic lesions. However, in both studies an error signaling procedure was used. This procedure has some disadvantages, e.g., patients who generally respond slower than healthy individuals might prefer to avoid additional button presses in between trials. Thus, they might notice their errors but miss to indicate them (cf. Wessel, 2012). However, significantly reduced Pe amplitudes in these patients provide additional evidence for impaired error awareness (Seifert et al., 2011). A further, previously unpublished analysis of the data by Seifert and colleagues broken down by lesioned thalamic subregions revealed that the Pe is absent in patients with lesions focused on the mediodorsal nucleus and only marginally reduced in amplitude in patients with focal lesions in the ventral anterior and ventrolateral anterior nuclei. Interestingly, the ERN showed the opposite pattern of impairment. Thus, it appears that the basal-ganglia-thalamocingulate circuit is involved in ERN generation, whereas the more arousal-related circuitry of the mediodorsal nucleus plays a role in error awareness and generation of the Pe. Given the limited sample size, further studies with thalamic patients are necessary that involve a procedure where participants are required to evaluate the accuracy of their response after every trial (e.g., as described in Klein et al., 2007a; Wessel et al., 2011).

### Posterior medial frontal cortex

There are mixed results with respect to the role of the pMFC in error awareness. While earlier studies did not find any difference in pMFC activity between perceived and unperceived errors (Hester et al., 2005; Klein et al., 2007a), recent studies did find a difference in medial frontal areas (Hester et al., 2009, 2012; Orr and Hester, 2012). Furthermore, recent electrophysiological studies on error awareness found larger ERN amplitudes in consciously perceived errors compared to unperceived errors (Scheffers and Coles, 2000; Steinhauser and Yeung, 2010; Wessel et al., 2011; Shalgi and Deouell, 2012; for a review see Wessel, 2012). While error correction appears to be affected by lesions of the pMFC (Swick and Turken, 2004; Modirrousta and Fellows, 2008), studies directly addressing error awareness in patients with pMFC lesions are lacking.

In sum, most error awareness studies identify the anterior inferior insula as crucial neuronal correlate of conscious error perception, but there is also preliminary evidence that the pMFC and thalamic regions are important structures for error awareness.

## Drugs affecting conscious error perception

It has been shown that the use of certain drugs attenuates the response of the aMCC to errors or diminishes the ERN. This has been demonstrated for cocaine, opioids, and alcohol (Ridderinkhof et al., 2002; Kaufman et al., 2003; Forman et al., 2004). Furthermore, the dopaminergic D2 receptor antagonist haloperidol reduces the ERN response to errors in flanker tasks (Zirnheld et al., 2004; De Bruijn et al., 2006), and there is evidence that smaller ERN amplitudes go along with reduced error awareness (see above). Moreover, subjects with lower D2 receptor density showed attenuated pMFC responses to negative feedback (Klein et al., 2007b). Therefore, the question arises whether the use of certain drugs also affects error awareness and, more specifically, whether dopamine (DA) plays a crucial role in conscious error detection. In the following, we will review those studies that investigated error awareness under pharmacological challenges.

Hester et al. (2007) showed that cocaine use can lead to reduced error awareness. They investigated a group of active cocaine users with the Go-NoGo error awareness task described above. Cocaine is assumed to exert its influence by blocking the re-uptake of DA, norepinephrine (NE) and serotonin and thereby increasing extracellular DA levels in those brain areas with afferents from the mesolimbic DA system (cf. Kuhar et al., 1991; Jocham et al., 2007). Thus, a long-term effect of cocaine use seems to be that the DA receptor density decreases (Volkow et al., 1990). This could explain an attenuated aMCC response in cocaine users (Kaufman et al., 2003; Klein et al., 2007b). However, error awareness in cocaine users was not decreased in general, but specific for certain error types. In the error awareness task employed by Hester et al. (2007), errors were committed when participants failed to withhold their response either to incongruent stimuli (first NoGo condition) or to the repetition of the same stimulus as in the trial before (repeat trials, second NoGo condition). Interestingly, cocaine users showed reduced error awareness only in repeat trial errors, but conscious error perception in incongruent trials was comparable to that in the control group. Therefore, one cannot unequivocally conclude that cocaine use leads to reduced error awareness. An alternative explanation could be that cocaine use might cause slight working memory or attentional impairments, and therefore, only repeat trial errors were noticed less often. Moreover, Garavan et al. (2008) showed that cocaine does affect insular activity. After i.v. cocaine administration, participants showed *enhanced* insular activity in response to performance errors. Note that an acute cocaine administration might evoke different effects than long-term cocaine usage.

In a later fMRI study using the same task, Hester et al. (2009) showed that error awareness is reduced in chronic cannabis users. In this study, reduced error awareness was associated with attenuated aMCC activity in cannabis users. There was also a relation between insula activity and error awareness: insula activity was negatively correlated with the amounts of cannabis used, and higher cannabis craving was correlated with less error awareness. Insula activations are reliably found in craving paradigms (Garavan, 2010). The craving for abused drugs seems to be linked to hypocretin (orexin) transmission in the insula, which in turn might modulate DA release in connected brain areas (Kenny, 2011). In mice, hypocretin has been associated with modulations in wakefulness, and it has been suggested that the hypocretin neural network might initiate arousal responses (Adamantidis et al., 2007), which would be a plausible adjustment after errors. It has been suggested before that errors might elicit an orienting response (Notebaert et al., 2009; Wessel et al., 2012), which is associated with increased arousal. Thus, hypocretin effects in the insula might be two-fold: on the one hand it could potentially increase arousal, and on the other hand, it could influence DA and NE release.

A recent study showed an enhancing effect of methylphenidate (MPH) on error awareness (Hester et al., 2012). Healthy participants perceived more errors consciously when they were under the influence of MPH than in the placebo condition. MPH has also proven to be effective in the treatment of cognitive deficits that can be observed after traumatic brain injury (Willmott and Ponsford, 2009), which have been associated with reduced awareness (O'Keeffe et al., 2004). Since MPH is a DA and NE reuptake inhibitor, it can be seen as indirect DA agonist (cf. Hester et al., 2012). Thus, this study provides further evidence that error awareness could be related to DA levels. Furthermore, a study by Frank et al. (2007) showed an effect of the catechol-o-methyltransferase (COMT) genotype on the Pe, which is systematically linked to error awareness. The val/met polymorphism of the COMT gene has been associated with prefrontal DA levels (Egan et al., 2001; Bilder et al., 2004).

Although the number of studies investigating neurochemical aspects of conscious error perception is very limited, there is converging evidence that the catecholamines DA and NE are highly relevant neurotransmitters associated with error awareness. Most direct evidence for a relation between these neurotransmitters and error awareness has been collected with psychostimulants that increase extracellular DA and NE. The role of hypocretin needs further investigation, but it seems to modulate DA, NE, and serotonin release as well. Given its proposed role in the orienting reflex and the generation of the P300 (and Pe) potentials (Nieuwenhuis et al., 2005), NE is likely to be involved in error awareness. Based on the current knowledge on the role of DA and NE in performance monitoring and attention (Aston-Jones and Cohen, 2005; Jocham and Ullsperger, 2009; Cools, 2011) one can assume that these neurotransmitters play a role in strengthening the error signal and enhance subsequent central neural and autonomic activity changes that contribute to conscious error perception. Differentiating the contributions of DA and NE to error awareness is an important goal for future research.

## Insula involvement in anosognosia for hemiplegia: a link to error awareness?

As already proposed by Vocat and Vuilleumier (2010), AHP and error unawareness might share some neuroanatomical correlates. Poor insight into the consequences of a neurological disease is related to poor treatment outcome and reduced treatment compliance. Sometimes anosognosia is also accompanied by a disturbance of the sense of agency and the sense of limb ownership (Karnath and Baier, 2010). In general, anosognosia can be observed following both right and left hemispheric brain damage with some predominance of appearance following right hemispheric insult. In the acute phase of a neurological disease, anosognosia is observed quite often: In a meta-analysis of 27 studies a median of 26% of patients following right hemispheric and a median of 10% of patients following left hemispheric stroke showed signs of anosognosia (Jehkonen et al., 2006).

The insular cortex has often been associated with deficit awareness (Karnath et al., 2005; Prigatano, 2009; Craig, 2010). Other relevant brain areas in AHP are the prefrontal cortex, the inferior parietal lobe, the angular gyrus, the supramarginal gyrus, and the anterior temporal lobe (Prigatano and Shacter, 1991). Especially during the first days of acute illness, damage to the posterior insular is predictive for developing anosognosia. Vocat and colleagues showed that in patients with *sustained* unawareness frontal and parietal brain areas were also affected (Vocat et al., 2010).

There might be two subcomponents of error processing: an early component that is not dependent on any kind of proprioceptive feedback but solely based on the efference copy of the executed action, and a second component that is more about the evaluation of the error and potential adjustments to avoid future errors of a similar kind (see Vocat and Vuilleumier, 2010 for a similar account of deficit awareness). Similarly, Prigatano (2010) claims that self-awareness is necessary for performance monitoring. This self-referential information (provided by interoceptive awareness, Craig, 2010) has to be integrated with external information (supplied by exteroceptive awareness, Craig, 2010) in order to come up with an accurate view of the situation or the action and its outcome, respectively. It has been suggested that this integration takes place in the insular cortex (Craig, 2011). When the representation of internal or external information is corrupted (what might be the case following brain lesions), deficient decisions or profound problems in awareness of the outcome of a decision/action might be the consequence.

Although several brain areas have been discussed to play a role in AHP (for reviews on AHP in general see e.g., Vuilleumier, 2004; Vocat and Vuilleumier, 2010), the insular cortex seems to play a prominent role in deficit awareness. The complex connectivity patterns of anterior and posterior insular cortex might suggest that awareness in general is the product of a network of brain regions all providing different kinds of information which finally allow awareness for internal and external information (e.g., Vocat and Vuilleumier, 2010). However, studies directly linking symptoms of AHP to electrophysiological (Pe) or functional (fMRI) correlates of error awareness are missing so far.

## Psychiatric illness, the insular cortex, and error awareness

Psychiatric patients sometimes show a high degree of lack of insight into their psychiatric condition. Because lack of insight might be related to deficient monitoring processes and reduced self-awareness, we review several studies that investigated error monitoring (mostly electrophysiological correlates of error monitoring or error awareness) in psychiatric patients. Lack of insight is, for example, a frequent observation in patients suffering from schizophrenia. Other psychiatric diseases like attention-deficit hyperactivity disorder (ADHD) and autism spectrum disorders (ASD) might also lead to patients' insensitivity to negative action outcomes, thereby promoting reduced error awareness. Since the insular cortex seems to play a crucial role in error awareness and AHP, an explicit focus will be put on the potential role of this area in disease symptomatology. This does, of course, not imply that a potential insular pathology alone accounts for the psychiatric disease under discussion.

### Clinical symptoms

Misattribution of thoughts and events to external sources as a consequence of altered monitoring processes is one key symptom of schizophrenia (Frith, 1987; Ullsperger, 2006). At least 50% of schizophrenic patients are without awareness for their disorder (Pia and Tamietto, 2006). The question, whether or not error awareness is compromised in schizophrenia was directly addressed by Mathalon et al. (2002) using a task in which no error signaling response was required. They compared patients suffering from schizophrenia with healthy controls while both groups worked on a performance-monitoring task with concurrent EEG recordings. Compared to healthy controls, schizophrenic subjects (especially those with paranoid schizophrenia) showed smaller ERN and larger CRN (correct-related negativity) amplitudes, but no differences in the Pe amplitude and subsequent post-error slowing (PES) (Mathalon et al., 2002). The authors concluded that perception of an error is sufficient but not necessary for producing the ERN, but that it is necessary for producing the Pe. Deficits in self-monitoring as indexed by altered performance monitoring might underlie some of the positive symptoms observed in schizophrenia as Mathalon et al. (2002) suggest. Corollary discharge dysfunction in schizophrenia (Ford et al., 2001) could explain not only the sensory integration deficits but also misattributions of action outcomes and agency and therefore result in error awareness deficits.

Although schizophrenia affects various brain areas, we focus here on studies reporting changes in the insula. Cytoarchitectonic alterations in the inferior insular and enthorinal cortex were found by Jakob and Beckmann (1986) in a subsample of postmortem brains of schizophrenia patients. Glahn et al. (2008) extended these findings with a meta-analysis by showing that gray matter of schizophrenic patients is reduced in a number of brain areas one of which being the bilateral insular cortex. Volume reduction of the left insular cortex in schizophrenia has been shown several times (Crespo-Facorro et al., 2000; Kasai et al., 2003; Kim et al., 2003). Using surface based morphometry in 57 schizophrenic patients Palaniyappan et al. (2011) demonstrated an inverse relationship between right posterior insula structure and the degree of insight in schizophrenia. The authors concluded that the right posterior insula might be the basis for insight as it allows for interoceptive awareness and self-appraisal of emotional states within a functional network that comprises also distant brain areas. In an extensive review of the existing literature Wylie and Tregellas (2010) summarized the role of the insular cortex in schizophrenia symptomatology. They concluded that damage to the insula or damage to a greater network comprising insular cortex could underlie many of the sensory-integration deficits observed in schizophrenia (Wylie and Tregellas, 2010). In a recent paper Williamson and Allman (2012) review the role of different functional brain networks in schizophrenia. Incorporating studies using voxel-based morphometry (VBM), DWI, and resting state functional MRI they report different brain networks as potentially relevant for schizophrenia. Besides (mal-) functioning of a network related to directed effort (comprising superior anterior and posterior cingulate cortex, auditory cortex, and the hippocampus) also its (disturbed) interactions with a brain network related to representations of thoughts, feelings, and actions (with the frontal and temporal pole and the frontoinsular cortex) might be involved in the pathophysiology of schizophrenia.

ADHD is associated with abnormalities in response to performance errors (O'Connell et al., 2009). PES, which might be indirectly linked to error awareness, was found to be reduced in a large sample of ADHD children (Schachar et al., 2004).

O'Connell et al. (2009) investigated electrophysiological correlates of performance monitoring in adult ADHD subjects. While making more errors, the ADHD patients showed an attenuated early and late Pe following erroneous responses. Furthermore, these subjects showed reduced electrodermal reactivity to an error, thus suggesting that errors had less emotional relevance to them. Wiersema et al. (2009) corroborated the findings by O'Connell et al. in ADHD subjects by showing reduced Pe amplitudes which were correlated with ADHD symptoms, normal sized ERN and no differences in behavioral adaptation following an error. They concluded that early automatic error detection processes are not affected in ADHD, but that there are illness related differences in later evaluative processes.

Several other studies investigated error processing in children with ADHD. It has been shown that ADHD children committed twice as many errors as healthy controls and did not show post-error behavioral adaptations, like PES (Schachar et al., 2004; Wiersema et al., 2005). As in ADHD adults, ADHD children also showed reduced Pe amplitudes as compared to healthy controls (Wiersema et al., 2005; Zhang et al., 2009).

Patients suffering from ASD sometimes show perseverative behavior, which might be interpreted as a consequence of impaired performance monitoring. These patients might be less sensitive to the course or the outcome of their actions thereby having an increased risk to repeat behavior over and over again. A role for the insular cortex might be assumed in this disorder: Ebisch et al. (2011) showed in a group of 12–20-year-old adolescents with high-functioning ASD in resting state fMRI that functional connectivity between right anterior and bilateral posterior insula with different other brain regions (posterior: inferior and superior somatosensory cortices; anterior: amygdala) was diminished as compared to results from a control group. Di Martino et al. (2009) were able to show an increased likelihood for hypoactivation of right anterior insula in studies analyzing brain activity in social tasks with patients suffering from ASD. Santos et al. (2011) investigated the frontoinsular cortex (layer V) of children suffering from autism using a stereologic approach. They found a significantly higher ratio of VENs to pyramidal neurons in the frontoinsular cortex of autistic children (for a discussion of VEN in insular cortex see “Insula: Structure and Connectivity”). Furthermore, they found a trend for an increase of the total number of VENs in frontoinsular cortex in autistic patients compared to their respective controls. The authors interpret these findings in terms of a potential neuronal overpopulation which might finally lead to increased interoception sometimes being described as part of the autistic syndrome.

Sokhadze et al. (2010) confronted autistic children with a task suitable for investigating error processing (no error-signaling response required). They demonstrated that these children committed significantly more errors, showed a smaller ERN with a larger latency, a trend toward a reduction in Pe amplitude compared to controls and no signs of PES; they rather showed post-error-speeding. The authors discuss that these alterations in performance monitoring might lead to reduced error awareness thereby allowing no successful adaptation of behavior following an error. Furthermore, Vlamings et al. (2008) were able to show that children diagnosed with ASD showed a smaller ERN and Pe and reduced PES.

### Discussion: error awareness and psychiatric illness

Direct studies of error awareness in psychiatric patients are rare. There is indirect evidence for altered error awareness in schizophrenia, ADHD, and autism based on ERN and Pe amplitudes. In schizophrenia patients, the ERN seems to be diminished, but the Pe seems to be unaffected. Given that reduced ERN amplitudes have been associated with impaired error awareness (Wessel, 2012), but perhaps only when accompanied by reduced Pe amplitudes, it remains unclear whether error awareness is attenuated in schizophrenia. In contrast, in ADHD the Pe is reduced, whereas the ERN seems to be unaffected. Based on the results by Murphy et al. (2012), showing that Pe latency and amplitude predicts error awareness, this suggests that error awareness is compromised in ADHD. In ASD both the ERN and Pe are diminished, suggesting that error awareness is reduced in this disease. However, the conclusions on error awareness in psychiatric disorders are based on a very small number of studies, and further studies investigating error awareness directly with proper error awareness tasks are needed. Since the insular cortex has often been associated with error awareness, we reported evidence for changes in cytoarchitectonics, cortical volume, and functional connectivity in the insula of schizophrenia or ASD patients. Furthermore, a recent review by Menon and Uddin (2010) suggested a prominent role for the anterior insula acting together with the aMCC as a key player within a so-called salience network which identifies behaviorally relevant internal and external stimuli. The anterior insula is thereby not only thought to detect salient stimuli but also to initiate switches between other major brain networks (i.e., default mode network and central-executive network), modulate autonomic activity (via interplay with the posterior insula) and finally having direct access to the motor system via the aMCC. The authors conclude that alterations in the integrity of this salience network might underlie the symptomatology of different psychiatric disorders. Taken together it is not only the structural and functional integrity of the insular cortex per se that seems necessary for mental health but also intact structural and functional connectivity between insular cortex and other brain areas is needed for intact cognitive functioning. Whether structural or functional changes of insular cortex are also directly responsible for the alterations in electrophysiological indices of error awareness observed in patients suffering from either disease remains, however, speculative.

## Conclusions

Deficits in performance monitoring in general, and error awareness in particular, might result from different pathological changes in the brain. The anterior insula has been discussed as part of an attentional network, and activity in this part of the insula is related to error awareness, whereas more posterior insula areas represent sensorimotor processes. AHP has been described as deficit in the re-representation of sensorimotor processes or as disorder of awareness for motor deficits and can be observed after posterior insular lesions. The anterior and posterior parts of the insula are highly interconnected. Thus, the insular cortex could be a structural link between error awareness and awareness of deficits or changes due to neurological or psychiatric diseases. Craig (2011) suggested that the integration of interoceptive and exteroceptive information takes place in the insula. Similarly, Kurth et al. (2010, p. 519) described the integration of “[…]different qualities into a coherent experience of the world[…]” as one potential role of the insula. This integration might be disturbed in anosognosia and insight deficits. Although evidence is rather indirect yet, error awareness seems to be attenuated in schizophrenia, ADHD, and autism. Further studies are needed with respect to the underlying neurotransmitter systems involved in error awareness, but preliminary evidence indicates a prominent role of the dopaminergic system.

**Figure 2:**
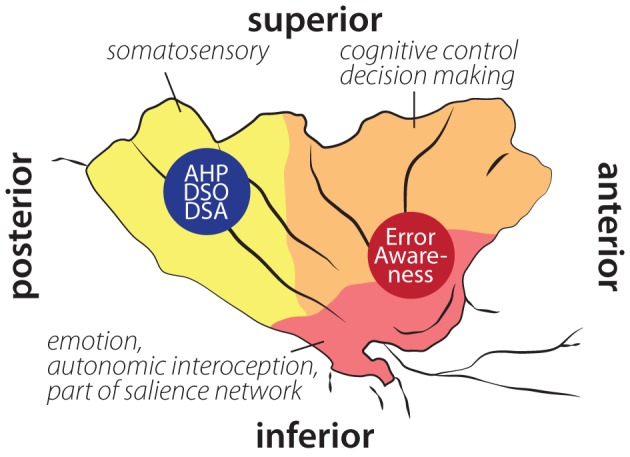
**Schematic illustration of insular cortex involvement in error awareness, anosognosia for hemiplegia (AHP), disturbed sense of ownership (DSO) and disturbed sense of agency (DSA) overlaid on schematic drawing of functional areas within the insula according to Deen et al. (2011).** Localization of AHP, DSO, and DSA based on Karnath et al. (2005); Baier and Karnath (2008); Karnath and Baier (2010). Localization of error awareness based on Klein et al. (2007a); Hester et al. (2009, 2012); Orr and Hester (2012).

In sum, the insula appears to receive and process information on surprising and unwanted states. The anterior insula is involved in (potential) problems with action performance, such as errors, unexpected outcomes (Wessel et al., 2012), or increased necessity of effort (Croxson et al., 2009; Prevost et al., 2010). The posterior insula seems more involved in integrating somatosensory input (see Figure 2). Hemiplegia results in unusual and erroneous somatosensory and proprioceptive feedback. An integrating feature of insular activity is that, if evidence for salient action course or salient body perception is high, it becomes active, is involved in the orienting response, and consequently in awareness. The finding that in AHP posterior insular cortex is affected supports this view—the salience of missing or distorted feedback from the hemiplegic limbs is not detected and processed appropriately. It remains to be tested, however, whether anterior insular lesions impair error awareness.

### Conflict of interest statement

The authors declare that the research was conducted in the absence of any commercial or financial relationships that could be construed as a potential conflict of interest.
